# Institution-to-Institution Mentoring to Build Capacity in 24 Local US Health Departments: Best Practices and Lessons Learned

**DOI:** 10.5888/pcd11.140017

**Published:** 2014-10-02

**Authors:** Maggie Veatch, Gail P. Goldstein, Rachel Sacks, Megan Lent, Gretchen Van Wye

**Affiliations:** Author Affiliations: Gail P. Goldstein, Bureau of Alcohol and Drug Use Prevention, Care and Treatment, New York City Department of Health and Mental Hygiene, Queens, New York; Rachel Sacks, Megan Lent, Bureau of Chronic Disease Prevention and Tobacco Control, New York City Department of Health and Mental Hygiene, Queens, New York; Gretchen Van Wye, Bureau of Vital Statistics, New York City Department of Health and Mental Hygiene, New York, New York.

## Abstract

**Introduction:**

Institutional mentoring may be a useful capacity-building model to support local health departments facing public health challenges. The New York City Department of Health and Mental Hygiene conducted a qualitative evaluation of an institutional mentoring program designed to increase capacity of health departments seeking to address chronic disease prevention. The mentoring program included 2 program models, a one-to-one model and a collaborative model, developed and implemented for 24 Communities Putting Prevention to Work grantee communities nationwide.

**Methods:**

We conducted semi-structured telephone interviews to assess grantees’ perspectives on the effectiveness of the mentoring program in supporting their work. Two interviews were conducted with key informants from each participating community. Three evaluators coded and analyzed data using ATLAS.ti software and using grounded theory to identify emerging themes.

**Results:**

We completed 90 interviews with 44 mentees. We identified 7 key program strengths: learning from the New York City health department’s experience, adapting resources to local needs, incorporating new approaches and sharing strategies, developing the mentor–mentee relationship, creating momentum for action, establishing regular communication, and encouraging peer interaction.

**Conclusion:**

Participants overwhelmingly indicated that the mentoring program’s key strengths improved their capacity to address chronic disease prevention in their communities. We recommend dissemination of the results achieved, emphasizing the need to adapt the institutional mentoring model to local needs to achieve successful outcomes. We also recommend future research to consider whether a hybrid programmatic model that includes regular one-on-one communication and in-person conferences could be used as a standard framework for institutional mentoring.

## Introduction

Institutional mentoring — an institution-to-institution learning framework using an interactive, facilitative process — may be a useful model for building the capacity of local health departments (LHDs) to address emerging public health challenges (1). By introducing a framework for in-person and remote interaction between an experienced, knowledgeable mentor institution and a group of mentee organizations, the institutional mentoring model incorporates training and technical assistance (T/TA) methods that have proven useful for LHD staff (2–7) in a peer learning, interactive, and supportive structure.

The Centers for Disease Control and Prevention (CDC) awarded the New York City Department of Health and Mental Hygiene (DOHMH) a Communities Putting Prevention to Work (CPPW) supplemental grant to develop an institutional mentoring program to support LHDs nationwide that are implementing new efforts to combat chronic disease (8). In this article, we report findings from an evaluation of mentees’ responses to the program and consider which elements were most effective in facilitating institutional capacity building.

## Methods

### Program background and description

Emerging public health challenges and epidemiological trends have led LHDs to shift focus from infectious to chronic diseases and from programs to policy, systems, and environmental change (9–11). Innovative methods that build on traditional areas of public health practice are needed to help LHDs develop effective approaches to these new challenges (11,12). For example, environmental interventions, which were essential strategies for controlling infectious disease in the last century, are being adapted to address chronic diseases today (12). However, LHDs need support and guidance to repurpose these foundational public health strategies to address challenges (10).

CDC developed the CPPW program to respond to this need for innovative, reinvigorated approaches to chronic disease prevention and control by funding 50 US communities to address obesity and tobacco use. Communities included cities, counties, tribal nations, and geographic regions (8).

From July 2010 to March 2011, DOHMH designed a mentoring program focused on CPPW objectives to address obesity and tobacco use in 4 topic areas: Built Environment, addressing obesity through community design; Breastfeeding, working in hospitals to increase breastfeeding initiation, duration, and exclusivity; Tobacco, reducing the use of and exposure to tobacco through media, policy, and coalition building; and School Food*,* improving school food quality. Because of its experience (12–15), DOHMH was well positioned to mentor other communities and developed a program that combined collaborative partnerships in an institutional mentoring framework to enhance LHD innovation and success.

Communities that received CDC CPPW funding to work in at least 1 of the 4 mentoring topic areas were invited by DOHMH to participate in the mentoring program. Of the 33 communities initially contacted, 24 committed to participate. The only exclusion criterion applied to the School Food topic; at least 1 school district per community was required to have 40,000 or more students. Mentees also had to agree to participate in the main activities of the program. Mentoring staff assessed each community’s needs to inform program development and model design. Participants from each community were staff members actively implementing programming, comprising a diverse group of entry-, mid-, and senior-level staff for each content area.

Two models were developed to address the diverse needs of the CPPW communities (Figure). The one-to-one model incorporated TA and coaching methods, and support was tailored to mentees’ individual needs. Its main strategy was a monthly DOHMH-led telephone call with each mentee. The call agenda focused on a topic selected by the mentee from a list prepared by DOHMH or identified by the mentee. DOHMH believed that the one-to-one model would be effective for topic areas in which a clear set of best practices had been established, including clinical guidelines, preventive service recommendations, and other replicable public health strategies. Examples include cigarette taxes (16) and baby-friendly strategies to increase breastfeeding (15); thus, the one-to-one model was used for the Tobacco and Breastfeeding content areas.

**Figure:**
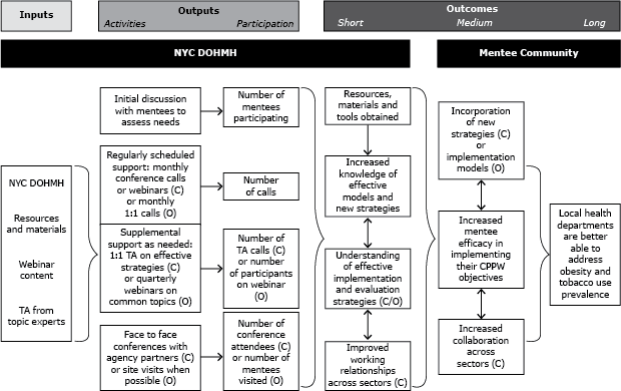
New York City Communities Putting Prevention to Work mentoring grant evaluation logic model, displaying the DOHMH planning process and results related to the 2 program model types used in the institutional mentoring program, the collaborative model, and the one-to-one model, 2010–2012. Abbreviation: NYC DOHMH, New York City Department of Health and Mental Hygiene; TA, technical assistance; C, collaborative model; O, one-to-one model.

The collaborative model used in-person conferences to facilitate interaction and network-building among mentees; it prioritized relationship building, strategy sharing, and collaborative problem solving to allow dynamic exchanges to foster innovation. In addition to 2 face-to-face conferences on each topic, DOHMH communicated at least monthly with mentees. The collaborative model also encouraged interagency collaboration, inviting stakeholders from nonhealth agencies to participate in the conferences and mentoring process. DOHMH believed this model would best accommodate the complex topics of Built Environment and School Food where no clear consensus on prescriptive action exists.

Participants in both models received monthly contact via webinars, conference calls, or e-mails; extensive resource guides; on-demand telephone support; and, in some cases, site visits during the 15-month project (January 2011 through March 2012). Contact between mentor and mentees typically occurred several times per month, depending on the pace of mentee project implementation and TA needs.

Four mentoring coordinators were hired to serve mentee needs in each program content area. Coordinators were midlevel professionals with experience in either content or TA provision. Senior DOHMH staff provided supervision and participated in TA calls, webinars, and conference calls.

### Evaluation

The mentoring program funded 2 DOHMH evaluators, who had no mentoring relationship with the communities, to assess program effectiveness through semistructured telephone interviews at the midpoint and close of the program. Midpoint interviews acted as proxy for baseline interviews that timing of the grant did not allow. Key informants who most directly participated in the program were interviewed. In most cases, the interview was with LHD employees; in communities in which CPPW work was subcontracted, a representative from that agency was interviewed.

An interview guide was developed to assess if and how DOHMH inputs affected mentees’ acquisition of resources, tools, understanding of effective implementation and evaluation strategies, and working relationships across sectors (Figure). The guide also explored how the program could be improved.

Interviews were recorded and transcribed. Responses were coded and analyzed separately by 3 evaluators by using Atlas.ti version 6.0 (Atlas.ti Scientific Software Development) to ensure that coding was consistent. Evaluators debriefed and engaged in member checks to ensure data quality. Grounded theory method was used to identify codes for emerging themes; the themes were grouped into main and subthemes (17). Because this was a program evaluation, institutional review board approval was not required.

## Results

Forty-four mentoring relationships were developed, drawn from 24 participating CPPW communities (Table). The average length for the midpoint interview was 52 minutes (range: 24–86 minutes); 45 interviews were completed because 1 community had 2 key informants who were interviewed separately because of scheduling. The interview at the grant’s end had a mean time of 25 minutes (range: 9–54 minutes); 45 interviews were conducted because 2 communities had 2 key informants who were interviewed separately because of scheduling and because 1 community dropped out.

**Table:** New York City Department of Health and Mental Hygiene Institutional Mentoring Program, Participants and Community (N = 24) Locations, by Topic Area, United States, 2010–2012

Community	One-to-One Model		Collaborative Model	
	Breastfeeding (n = 10)	Tobacco (n = 11)	School Food (n = 9)	Built Environment (n = 14)
Austin/Travis County, Texas		X		
Boston, Massachusetts	X	X		X
The Cherokee Nation		X		X
Chicago, Illinois	X			X
Suburban Cook County, Illinois	X			X
Douglas County, Nebraska			X	X
Jefferson County, Alabama		X	X	X
Los Angeles County, California	X		X	
Louisville, Kentucky	X		X	X
Miami-Dade County, Florida	X		X	X
Mid-Ohio Valley, West Virginia	X			
Mobile County, Alabama		X		
Multnomah County, Oregon				X
Nashville and Davidson County, Tennessee	X		X	X
New York, New York	X			
Philadelphia, Pennsylvania	X	X	X	X
Pima County, Arizona				X
Providence, Rhode Island		X		
County of San Diego, California				X
Florence County, South Carolina		X		
Horry County, South Carolina		X		
King County, Washington		X	X	X
Clark County, Nevada			X	
Washington, DC		X		

Five key program strengths emerged from the interview data; 1 additional strength was specific to each model type. Several program limitations also surfaced. Per grounded theory, identified themes carry equal weight; the order in which themes are presented does not reflect their level of importance.

### Program strengths for both model types

#### 1. Learning from DOHMH’s real-world experience

Receiving TA grounded in real-world experience was cited by participants in both model types as a key aspect of the mentoring program. DOHMH’s experience implementing similar initiatives allowed mentors to provide advice and insight:

[Mentoring] was an opportunity to learn from someone who had gone . . . down this path before that could help us to identify the best practices. I think we would have gotten there, it just made our travel a little easier. — Breastfeeding participant

Mentees often knew what they wanted to do but struggled with implementation. Therefore, DOHMH included practical details of implementation, such as where to place signage to encourage walking. One respondent described how this support rendered institutional mentoring more useful than T/TA programs in which an expert provides theoretical guidance:

TA providers can provide . . . examples of what happened in other communities, but they are not frequently the actual implementers. They can’t say, “here is how we did it, and this is what worked and didn’t work.” We can’t get that from . . . TA providers, but we can get that from the [mentors]. — Tobacco participant

DOHMH also helped communities navigate bureaucracies and frame issues, supporting consensus building and improving overall efficiency. For instance, mentors in the area of Breastfeeding encouraged practitioners who were accustomed to promoting breastfeeding’s health benefits to emphasize the business case. Mentors in the area of Built Environment coached LHDs working with planning and transportation agencies to detail how integrating health considerations into urban planning could complement their work.

#### 2. Adapting proven materials and methods to local needs

DOHMH provided sample language for various written products, including requests for proposals, presentations, fact sheets, job descriptions, television advertisements, radio scripts, Facebook pages, and data collection tools. DOHMH also provided strategies for identifying and engaging key stakeholders, and detailed descriptions of policy implementation. Although some of these resources were available elsewhere, the mentoring program provided easy, organized access, allowing mentees to work more efficiently:

When we [gave mini-grants to coalition members], we took 95% of the text from the New York City RFP so that we didn’t have to reinvent the wheel. — Tobacco participant

#### 3. Incorporating new approaches and sharing strategies

Sharing new ideas and learning about innovative strategies helped mentees to broaden their perception of what they could accomplish:

Even if it’s not something we can do immediately, [mentoring] helped us plant the seeds to get to where we need to go. I look at it as an investment that will pay dividends in the future. — School Food participant

Among the Built Environment mentees, 1 LHD initially conceived of its project as a limited effort focused on allowing community members to use schools for exercise outside school hours. After attending the mentoring conference, the mentee expanded the project scope to include a community walkability assessment, altering building plans to increase physical activity, and collaborating with the local transportation department. Communities also learned strategies to demonstrate the impact of their activities, such as collecting data on bicycle and pedestrian use of roads to measure the effect of improved streets for nonmotorists.

#### 4. Developing the mentor–mentee relationship

Nearly all mentees noted that the ongoing mentoring relationship helped them feel more inclined to approach DOHMH for support.

It was a little more natural to follow up with [the mentoring coordinator] . . . as opposed to trying to educate somebody else about what we need. She already had that background so she knew what we needed. — Built Environment participant

Several communities reported that they preferred mentoring to T/TA programs because, when accessing help, they did not need to provide background and context because of the existing relationship with the mentor:

People are more likely to reach out and ask for help from friends, especially if it’s someone who has experience and . . . the ability to communicate . . . effectively to you. Establishing a relationship and a sense of trust between two entities makes a difference. — Tobacco participant

Additionally, DOHMH was familiar with mentees’ needs and was able to respond to them quickly and effectively. One mentee noted:

I could have spent hours on researching certain questions, [but by] just giving them a call they were able to just give me the answer over the phone. — School Food participant

#### 5. Creating momentum

Mentees in both models felt the mentoring program increased their momentum. Several participants reported that mentoring provided reassurance that they were “on the right track,” further motivating them:

A lot of people see New York City as being at the forefront of . . . smoke-free air laws, and to hear that they were at the same place we were not too terribly long ago, fighting some of the same battles that we’re fighting . . . provides a lot of encouragement. — Tobacco participant

The collaborative model appeared particularly useful in building mentees’ confidence and allowing them to feel connected to a larger body of work and a nationwide movement:

It has inspired more passion toward the built environment work and the possibilities. It has forced us to accelerate the work that we do. And because of that, it actually facilitated the health impact assessment training that we had this week, because we were able to make connections with city departments earlier and quicker. — Built Environment participant

### Program strengths specific to a single model type

#### 1. Establishing regular communication (one-to-one model)

Mentees participating in the one-to-one model identified regular communication as beneficial in facilitating the flow of information from mentor to mentee. Monthly calls with individual mentees allowed DOHMH to anticipate obstacles and tailor TA that was responsive to each community’s needs. This structure worked well, because mentees were not always sure what questions to ask; a mentee noted that “a lot of times you don’t know what’s helpful until you receive it.” Some mentees cited validation from DOHMH, as an expert in the field, as a benefit of the one-to-one relationship.

#### 2. Encouraging face-to-face peer interaction and learning (collaborative model)

In the collaborative model, mentees often described face-to-face time at the conferences as instrumental to peer learning and network building. Mentees were required to bring local partners from other agencies to mentoring conferences. As a result, they noted that attending the conferences together helped to foster peer learning across and within agencies:

Being in a room with the [school] district automatically builds better rapport, better connection with your colleagues. We hadn’t spent that much time with the representative from the district so . . . the time . . . to network and to even just sit down and eat lunch with the district . . . that in itself fosters a stronger relationship. — School Food participant

In-person interaction enabled LHDs to better understand the challenges and limitations of other agencies’ work, and partnering agencies learned how their work could influence health outcomes:

[Non-LHD participants] realized that it’s not just public health that could impact the well-being of the community . . . they became champions. . . . And, everything’s been easy from there because they . . . realized that they have power and an important role. — Built Environment participant

Furthermore, relationships in the collaborative model were built across mentee communities. As a result, networks were developed that helped mentees to explore promising practices for topics for which a standard approach has not yet been developed.

### Improving on the model

The most common suggestion for improvement from mentees across both model types was that access to mentoring earlier, particularly while developing grant objectives, would have been useful. In the one-to-one model, mentees desired more opportunities for peer network building through in-person contact with other communities. Collaborative model participants expressed interest in visiting New York City to learn about programming first-hand, and those mentees were brought to visit. In addition, during the midpoint evaluations, several mentees reported that large group conference calls were helpful but also intimidating and difficult for everyone to share. Smaller calls were subsequently implemented. Communities with more evolved built environment initiatives reported deriving less benefit from the mentoring program than communities with newer initiatives. Many communities commented that they would benefit most from working with similar communities, because of the variation in the structure of government in communities across the nation.

Among mentees participating in the collaborative model, the most commonly cited limitation was the need for more individual attention, and they also expressed a desire for site visits. Mentees in the collaborative model also desired even more face-to-face conferences and opportunities for peer interaction.

## Discussion

Mentoring program participants overwhelmingly indicated that working with DOHMH improved their capacity to achieve their CPPW objectives. In both models, key strengths of the institutional mentoring framework included DOHMH’s provision of practical advice, resources, opportunities for sharing ideas, and the development of mentor–mentee relationships and peer learning networks.

Several differences between the models emerged. Regular, tailored communication about standard guidelines and best practices, from mentor to mentee, was critical to success in the one-to-one model. By contrast, peer learning networks in the collaborative model helped mentees explore promising approaches for new topics. Mentees noted these networks to be among the most beneficial outcomes of mentoring. Another difference between the models was related to the mechanisms through which each built a feeling of support among mentees. LHDs and other institutions participating in the one-to-one model often framed support in terms of validation from DOHMH, while participants in the collaborative model reported feeling inspired by their peers and sensing that they were part of a movement. Finally, participants in the collaborative model noted that they would have preferred more individual attention from DOHMH, while participants in the one-to-one model said they would have benefited from access to a peer learning network.

Because topic areas were different, direct comparison of the models was difficult. However, on the basis of these findings, an ideal model for institutional mentoring may be a hybrid model that features regular one-on-one communication between mentor and mentees as well as in-person conferences that facilitate peer networking, which would integrate the benefits of formal quality improvement collaboratives with tailored T/TA programs and coaching opportunities.

This study has limitations. We did not engage an independent evaluator for this evaluation process. Although interviewers had no relationship with the communities, respondents may have been hesitant to share negative views of the program with DOHMH interviewers. In addition, because we did not compare communities that did not receive mentoring, we cannot relate mentees’ positive impressions of the program structure to concrete project achievements. We also did not assess education levels and experience of participants, and this lack of assessment may have affected their experience in the process. Our results are consistent with those of other studies of collaborative and peer learning networks (3).

Finally, although results related to specific content have been included in 2 published articles related to individual program areas (12,15), they were excluded as being beyond the scope of this analysis, which was focused on the program structure.

Mentees’ positive response to the institutional mentoring framework indicates that both the one-to-one model and the collaborative model were effective in supporting CPPW grantees. Although further evaluation is needed, our results are promising and merit further investigation. We encourage evaluators to assess whether a hybrid model that includes regular one-on-one communication as well as in-person conferences that facilitate networking could be effectively used as a framework for institutional mentoring.
